# An update on visual prosthesis

**DOI:** 10.1186/s40942-023-00498-1

**Published:** 2023-11-23

**Authors:** Kailyn A. Ramirez, Laura E. Drew-Bear, Maria Vega-Garces, Henry Betancourt-Belandria, J. Fernando Arevalo

**Affiliations:** 1 Rutgers Robert Wood Johnson Medical School, New Brunswick, NJ 08901 USA; 2 The Wilmer Eye Institute, Johns Hopkins University School of Medicine, 600 N. Wolfe Street, Maumenee 713, Baltimore, MD 21287 USA; 3 Rutgers New Jersey Medical School, Newark, NJ 07107 USA; 4 UF Health Cardiovascular Center, University of Florida, Jacksonville, FL 32209 USA

**Keywords:** Artificial vision, Retinal prostheses, Retinitis pigmentosa, Stimulation, Visual prostheses

## Abstract

**Purpose:**

To review the available evidence on the different retinal and visual prostheses for patients with retinitis pigmentosa and new implants for other indications including dry age-related macular degeneration.

**Methods:**

The PubMed, GoogleScholar, ScienceDirect, and ClinicalTrials databases were the main resources used to conduct the medical literature search. An extensive search was performed to identify relevant articles concerning the worldwide advances in retinal prosthesis, clinical trials, status of devices and potential future directions up to December 2022.

**Results:**

Thirteen devices were found to be current and were ordered by stimulation location. Six have active clinical trials. Four have been discontinued, including the Alpha IMS, Alpha AMS, IRIS II, and ARGUS II which had FDA and CE mark approval. Future directions will be presented in the review.

**Conclusion:**

This review provides an update of retinal prosthetic devices, both current and discontinued. While some devices have achieved visual perception in animals and/or humans, the main issues impeding the commercialization of these devices include: increased length of time to observe outcomes, difficulties in finding validated meaures for use in studies, unknown long-term effects, lack of funding, and a low amount of patients simultaneously diagnosed with RP lacking other comorbid conditions. The ARGUS II did get FDA and CE mark approval so it was deemed safe and also effective. However, the company became more focused on a visual cortical implant. Future efforts are headed towards more biocompatible, safe, and efficacious devices.

## Introduction

**Fig. 1:**
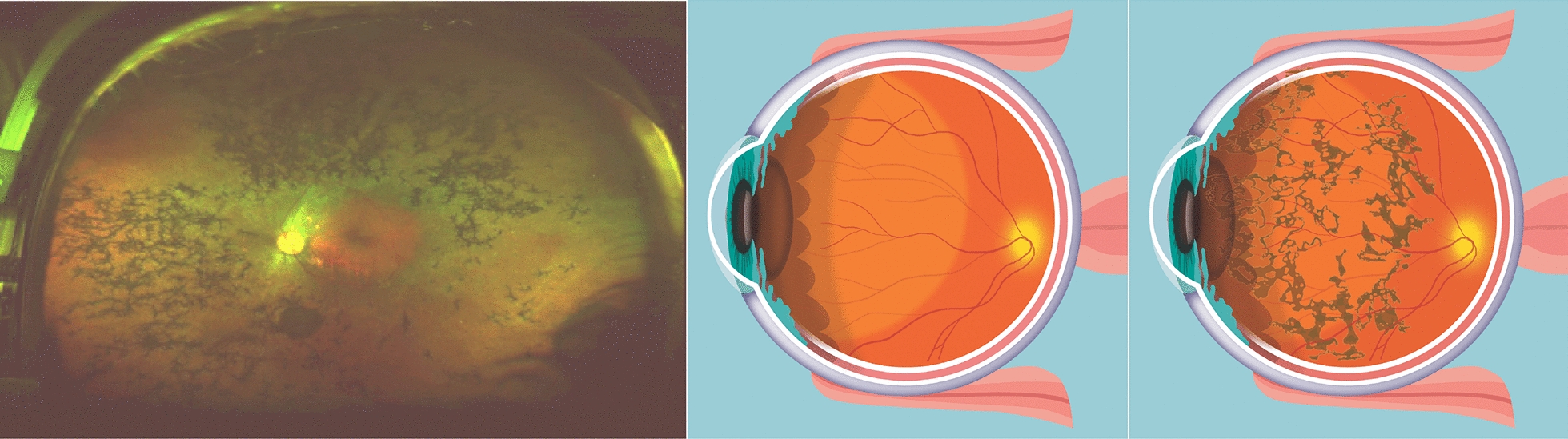
RP is a group of genetic progressive diseases, with an estimated prevalence of 1 in 4000 in the United States, that leads to total blindness. Though RP can be caused by mutations in any of over 190 genes, all lead to degeneration of the photoreceptor layer of the retina. The relative preservation of inner retina has led to efforts to develop retinal prostheses to stimulate residual surviving tissue. **Left**: the classic clinical triad of RP is arteriolar attenuation, retinal pigmentary changes (could be either hypopigmentation and/or hyperpigmentation in form of bone-spicule and pigment clumpings), and waxy disc pallor. **Middle cartoon**: normal eye. **Right cartoon**: eye with RP

Retinitis Pigmentosa (RP) is a class of rare inherited diseases leading to retinal degeneration (Fig. 1) over time [1]*.* This degeneration promotes photoreceptor cell death and retinal pigment epithelium (RPE) atrophy [2]. Despite irreversible loss and degeneration of photoreceptors, the nerve fiber and inner retinal neuronal cells of patients with RP remain largely preserved [3]. Patients diagnosed with RP progressively lose night and peripheral vision, leading to a narrowed visual field and remarkably diminished vision. As of February 2021, about 82,500 to 110,000 people in the United States have been diagnosed with RP or a related disorder [4]*.* Due to the variable presentation of this disease, some patients may experience significant visual loss in childhood while others may be left asymptomatic well into adulthood. Given the incurable nature of this condition, historically, there have been no recognized treatments administered to patients diagnosed with the disease. With recent advancements in gene therapy and artificial vision prosthetics, patients living with the disease may have a chance to partially regain sight.

Vision prosthetics are relatively new to the market with breakthroughs in research regarding electrical stimulation of the visual cortex dating as far back as 1929 [5]. These devices retain the potential to enhance patient quality of life and provide patients with a sense of independence as further developments persist into the near future. The devices currently being tested transduce light into electrical signals that are then transmitted right onto remaining retinal tissue, the optic nerve, or the occipital visual cortex in the brain. Many devices utilize an electrode system in which it is theorized that the quantity of these electrodes is directly correlated to increased vision restoration. Currently these devices have shown that otherwise blind patients are able to sense motion, locate objects, follow a path and recognize large letters.

This review serves to provide current information on the history and ongoing status of retinal prostheses, clinical trials, gaps in research, future directions for vision prostheses and new implants.

## Methods

A literature review was performed using PubMed, GoogleScholar, MedLine, IEEExplore, ScienceDirect, and ClinicalTrials.gov through November 2022. The following keywords were used: ‘‘retinitis pigmentosa”, ‘‘artificial vision”, ‘‘ARGUS II”, ‘‘bionic eye”, ‘‘retinal prosthesis”, ‘‘retinal implant”, ‘‘epiretinal stimulation”, ‘‘subretinal stimulation”, ‘‘suprachoroidal stimulation”, ‘‘optic nerve stimulation”, ‘‘optic nerve prostheses”, ‘‘occipital lobe prostheses”, ‘‘occipital lobe stimulation”, ‘‘cortical visual prosthesis”, ‘‘cortical stimulation”. Of the studies retrieved, we reviewed all English publications. Reference lists of the analyzed articles were also considered as a potential source of information.

## Results

Several devices currently exist and are undergoing trials to study their efficacy and longevity. These devices utilize various parts of the visual pathway to assist patients regain some light perception. Most devices are surgically placed epiretinal (on the retinal surface and adjacent to the retinal ganglion cell layer) or subretinal (under the adjacent retina or in place of remnants of the retinal pigment epithelial and photoreceptor layers) [7]. Some designs allow for retinal neurons to be directly excited to elicit an electrical stimulus discerned by patients [6]. Other designs require the placement of devices between the choroid and the sclera, or on the exterior of the sclera. Some devices bypass the eye anatomy completely, directly stimulating the visual cortex to generate visual signals. Such advances in research and technology that have allowed for the bypass of multiple areas of the visual pathway support further investigation of the efficacy of these devices in treatment of a wider range of vision threatening diseases not limited to RP.

(See Table 1: Visual prosthesis summary).

**Table 1:** Visual prosthesis summary

Device name	Company/research consortium	Array location	Device stage	Clinical trial identifiers/status
NR600 System	Nano Retina, Israel (company)	Epiretinal	Clinical	NCT04295304 (recruiting)
IMIE 256	Golden Eye Bionic, USA and IntelliMicro Medical, China (companies)	Epiretinal	Clinical	None
POLYRETINA	Diego Ghezzi research team (École Polytechnique Fédérale de Lausanne)	Epiretinal	Pre-clinical	None
EPI-RET3	RWTH Aachen	Epiretinal	Clinical	None
PRIMA	Pixium Vision, France (company)	Subretinal	Clinical	NCT03392324, NCT04676854 (recruiting) NCT03333954 (active, not recruiting)
IMTC’s HARP4k Retinal Prosthesis System	Iridium Medical Technology, Taiwan (company)	Subretinal	Pre-clinical	None
Gen 2 suprachoroidal device	Bionic Vision Australia (Research consortium)	Suprachoroidal	Clinical	NCT03406416 (completed), NCT05158049 (enrolling by invitation)
Phoenix-99	Bionic Vision Australia (Research consortium)	Suprachoroidal	Pre-clinical	None
STS	Osaka University, Japan (Research consortium)	Intrascleral	Pre-clinical	None
ORION	Second Sight Medical Products, USA (company)	Occipital lobe	Clinical	NCT03344848 (active, not recruiting)
CORTIVIS	Biomedical Technologies, Spain (company)	Occipital lobe	Clinical	NCT02983370 (recruiting)
ICVP	Illinois institute of technology	Visual cortex	Clinical	NCT04634383 (active, recruiting)
AV-DONE	NIDEK CO, Japan (company)	Optic nerve	Pre-clinical	None

### Epiretinal devices

The general constituents of epiretinal devices include an external and an implanted component. The external component is composed of a camera, signal processor, power and data transmitter. The implanted unit includes a series of stimulatory electrodes, a stimulator and a power and data receiver. Captured images from the camera are modified into digital data and further modified within the system to create an electrical stimulus in which the electrodes use to transmit impulses onto the remaining intact retinal tissue. These prostheses interface directly on the ganglion cell layer [7]. Epiretinal designs benefit from being comparatively less complicated and retaining lower levels of risk during implantation [8, 9] With this being the case, most of the efforts in advancing retinal prosthetics have been placed on epiretinal devices. (See Table 2: Epiretinal device characteristics).

**Table 2:** Epiretinal device characteristics

Device	Electrode specifications	Implant Size	Benefits	Reported SAE/AE’s	Clinical Status
NR600	600 3D microelectrodes (typical length of 150 ± 30 µm, and a maximum exposed tip height of 50 µm)	Three different lengths available varying between 20 and 26 mm in axial lengths	- Porous and chemically stable electrode material - Passivation layer to minimize shunting currents and limit electrode active area stimulation - Configurable settings - Lower peak intensity - No need for surplus wiring outside the eye - Relatively low risk procedure - Fast healing and recovery time	- Mild corneal edema along with slightly elevated intra-ocular pressure - Intra ocular lens luxation with subsequent elevated IOP	In clinical trials available in Italy, Israel, and Belgium
IMIE 256	256 electrodes: two sizes of disc-shaped electrode diameters including 248 large electrodes (210 µm in diameter) and 8 smaller electrodes (160 µm in diameter)	2 sizes: Area covers 4.75 mm × 6.50 mm center-to-center pitch is 350 µm for the large electrodes and 300 µm for the small electrodes	- Reduced risk of episcleral electronics capsule exposure - Greater density of functioning electrodes - 100% of subjects implanted performed better on visual tests with the system on versus off. - Compact size matching retinal curvature - Low power consumption - Reduced dissipated heat to surrounding tissue - Multiple layers of biocompatible and durable barrier material to withstand bodily fluid corrosion - Improved manufacturability for mass production allowing for future reduced costs [1	- Device displaced slightly toward the upper temporal position in the macula - low intraocular pressure in the implanted eye 6 weeks after repositioning	Future clinical trials for testing in more patients over a longer follow-up period
POLYRETINA	2215 stimulating pixels (80 and 130 µm diameter)	24 mm axially × 24 mm laterally × 14 mm wide	- Extra layer of PDMS substrate material to reduce strain - Foldable for implantation ease - Hemisphere shape matching retinal curvature - Reduced retinal tissue heating - Homogeneous temperature distribution during deposition - Large diameter of photovoltaic electrodes - Preserved high deformability - low level of acute immune response activation	Information not available	In vivo study showed that it restores light-evoked cortical responses at safe irradiance levels and is tolerable after two weeks if implantation
EPI-RET3	25 electrodes (100 µm diameter)	40 mm length × 3 mm width	- Wireless implant - Short surgical time - Direct electrode to retinal stimulation	- Four cases (three mild and one moderate) of non-progressive epiretinal gliosis at the tack fixation site - Inflammatory reaction in the implanted eye due to corneal sutures - Slight decline in residual visual perception in two patients post implant removal of the implanted eye - Minor choroidal atrophy within the area where the retinal tacks had been placed - removal of the implant had caused a retinal break (treated with laser coagulation and silicone oil tamponade) - acute sterile hypopyon after implantation - a thick epiretinal membrane in the tack area at the retinal center	Six patients were implanted for 28 days. No active clinical trials at this time

The *NanoRetina 600 (NR600) System* consists of two main components: a miniature implanted device and a pair of eyeglasses. The eye glasses are worn to power the implant and provide a clear image to pass with minimal distortion [10]. The implant is surgically placed and uses a three dimensional (3D) neural interface technology, with a lower optimal energy level that results in improved safety and higher specificity [11]. The procedure for implantation of the device is said to be low risk with a fast healing and recovery time due to the implant containing all necessary functionalities, reducing the need for surplus wiring outside of the eye globe. Additionally, the NR600 encompasses an increased number of active electrodes that are closely spaced to each other, providing a higher resolution image. Patients with the device are able to fine-tune different light settings and calibrate the stimulation parameters to suit their individual needs. Recruitment for clinical trials is currently ongoing. The study began as of January 17th, 2020, and is expected to continue into June of 2023. Currently, trials are only being offered in Italy, Israel, and Belgium and do not have FDA approval.

**Fig. 2:**
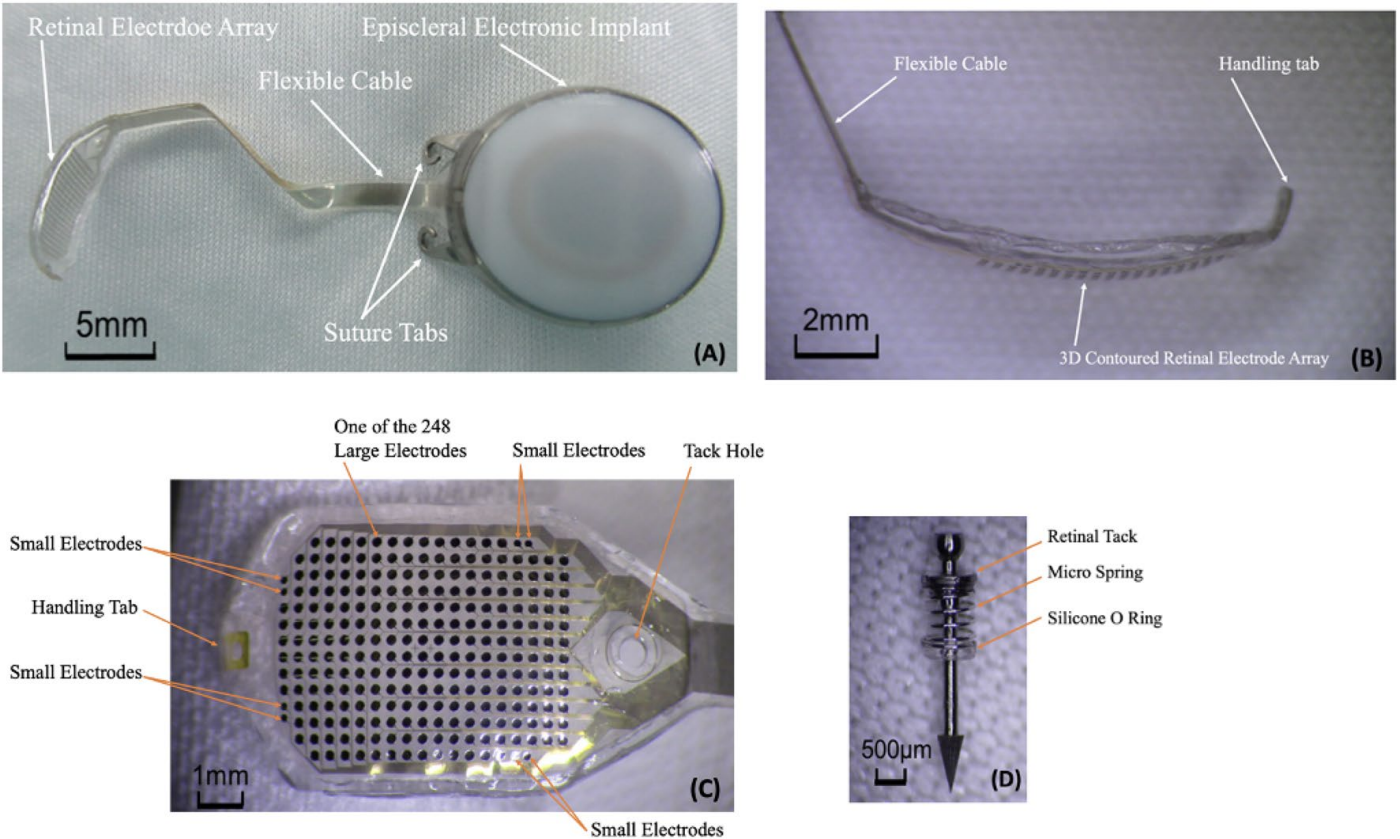
Internal component of the IMIE 256 (Modified and reprinted with permission from Xu H, Humayun MS, et al. First Human Results With the 256 Channel Intelligent Micro Implant Eye (IMIE 256). Transl Vis Sci Technol. 2021 Aug 12;10(10):14.)

**Fig. 3:**
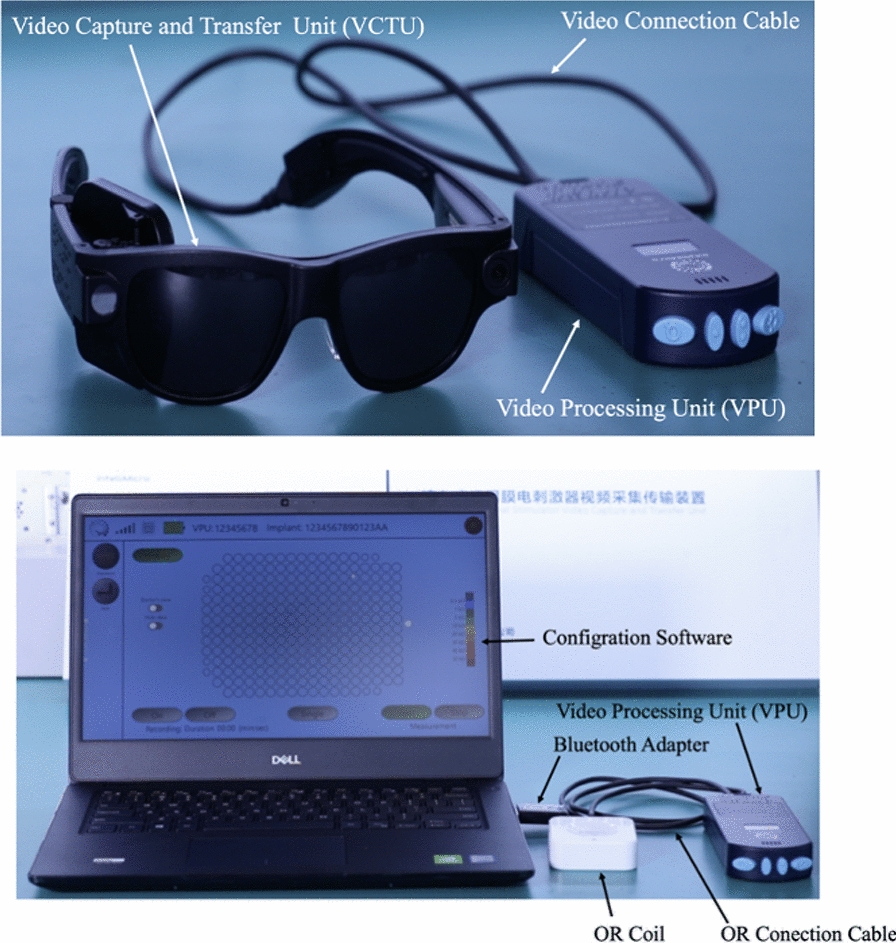
External component of the IMIE 256 (Modified and reprinted with permission from Xu H, Humayun MS, et al. First Human Results With the 256 Channel Intelligent Micro Implant Eye (IMIE 256). Transl Vis Sci Technol. 2021 Aug 12;10(10):14.)

The *256 Channel Intelligent Micro Implant Eye (IMIE 256)* also known as the Theia α Implantable Retinal Stimulator, also consists of an internal (Fig. 2) and external component (Fig. 3). The internal portion includes an episcleral electronic implant, a trans-scleral microfabricated cable and a custom contoured retinal electrode array consisting of a total of 256 electrodes [12]. The external portion contain a video capture and transfer unit (VCTU), a video processing unit (VPU), and a configuration/fitting system. First human clinical trials implanted the device into the right eyes of five subjects with end-stage RP yielding no complications. Subjects underwent visual rehabilitation for 90 days and their visual performance was evaluated using the grating visual acuity test, Tumbling E visual acuity test, direction of motion, square localization, and orientation and mobility test. Subjects who had the opportunity to complete all of these evaluations were significantly able to complete the tasks successfully with the device on compared to when the device was turned off. Currently, future clinical trials are underway in a larger patient sample size, who are expected to have a longer follow-up period.

*POLYRETINA* is a photovoltaic wide field epiretinal prosthesis based on polydimethylsiloxane (PDMS). The use of PDMS in POLYRETINA is said to be largely due to its transparency, elasticity, low Young’s modulus, and high strain to failure[13]. This device is foldable in nature, allowing for ease in implantation through a small scleral incision. The hemispherical shape of the device also matches the curvature of the eye allowing for full coverage of the retinal surface. A key benefit regarding the design of the device is that it lacks cytotoxicity while respecting optical and thermal safety standards. Pre-clinical studies have demonstrated a lifetime of at least 2 years [13]. Furthermore, results obtained ex vivo with retinal tissue explanted from a mouse model of RP demonstrated the ability to activate retinal ganglion cells (RGCs) at a safe irradiance level with a high spatial resolution, equivalent to the electrode pitch (120 μm) [13, 14]. An in vivo assessment of *POLYRETINA* in blind Göttingen minipigs showed that it restored light-evoked cortical responses at safe irradiance levels and is tolerable after two weeks of implantation [14].

The *EPI-RET 3* has both internal and external components with the internal being solely intraocular. An key design feature is that its body is coated with parylene C to ensure biocompatibility while the electrodes are sputter coated with iridium oxide to maximize the charge-delivery capacity [15]. The external component comprises an external camera and a visual processor that wirelessly transmits the calculated spatiotemporal patterns of stimulation pulses to the internal component using ultrahigh-frequency-pulsed charge-controlled stimulation. This reduces artifacts and allows bidirectional stimulation and recording by the microelectrodes [16–18]. The unique feature that allows EPIRET3 to stand apart from other epiretinal devices is its ability to obtain energy or data via inductive links. This makes the use of a physical transscleral cable unnecessary, reducing risk of erosion or infection. During clinical trials, the implants were not allowed to remain in the participants eyes for periods exceeding 1 month by request of the ethics committee since they were considered experimental devices. Therefore, the devices were removed at the end of the acute testing period. However, during the study, the visual acuity experienced by the study subjects ranged from no light perception to hand movements [15].

### Subretinal devices

In contrast to the epiretinal configuration, the subretinal configuration consists of an implant positioned behind the retina in place of the photoreceptors [7]. Despite the ease of surgical insertion of the epiretinal implant, subretinal designs benefit from utilizing intact medial retinal processing pathways made up of amacrine, horizontal, and bipolar cells. As with any surgery, subretinal implantation does not go without its risks. These risks included but were not limited to: increased intraocular pressure, damage to the conjunctiva, and damage to the already existing retina such as detachment and/or hemorrhage. [See Table 3: Subretinal device characteristics].

**Table 3:** Subretinal device characteristics

Device	Electrode specifications	Size	Advantages	SAE/AEs	Clinical status
PRIMA	378 electrodes (100 µm in width)	2 × 2 mm and 30 microns thick	- May result in natural conversion of pulsed spatiotemporal stimulation pattern into bursts of spikes from the retinal ganglion cells - Minimally invasive procedure - Targeted electrical stimulation	- Reduced retinal thickness	In clinical trial for age-related macular degeneration (France and USA)
IMTC’s HARP4k retinal prosthesis system	4000 microelectrodes (30 µm thick)	30 mm	- Contact lens shape	Information not available	Designed for retinitis pigmentosa and age-related macular degeneration
			- Wireless		
			- Expected to support face recognition and reading font size > 14 pt		
			- High granularity and efficiency		
			- Biocompatible and implantable		
			- Flexible technology interfacing soft tissue		

**Fig. 4:**
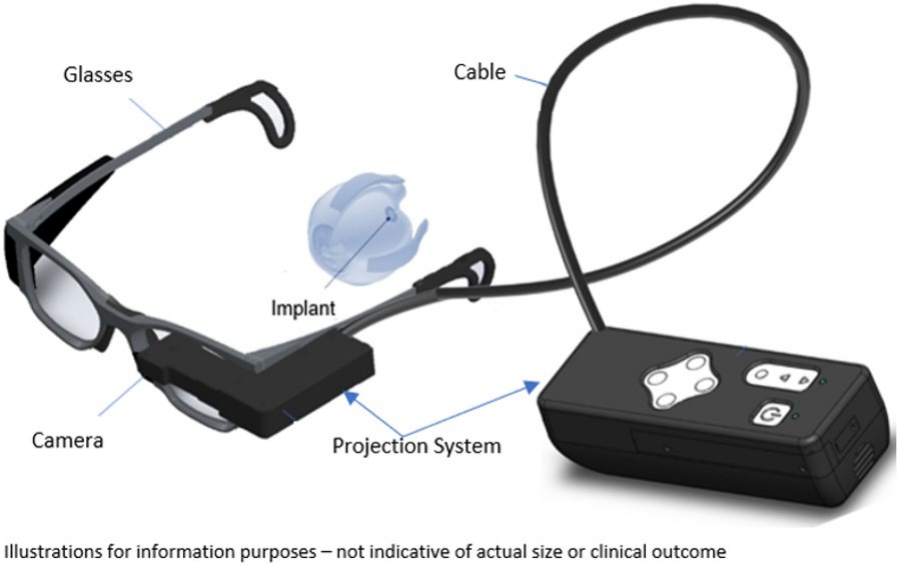
PRIMA Device (Courtesy of PIXIUM VISION, Paris, France)

**Fig. 5:**
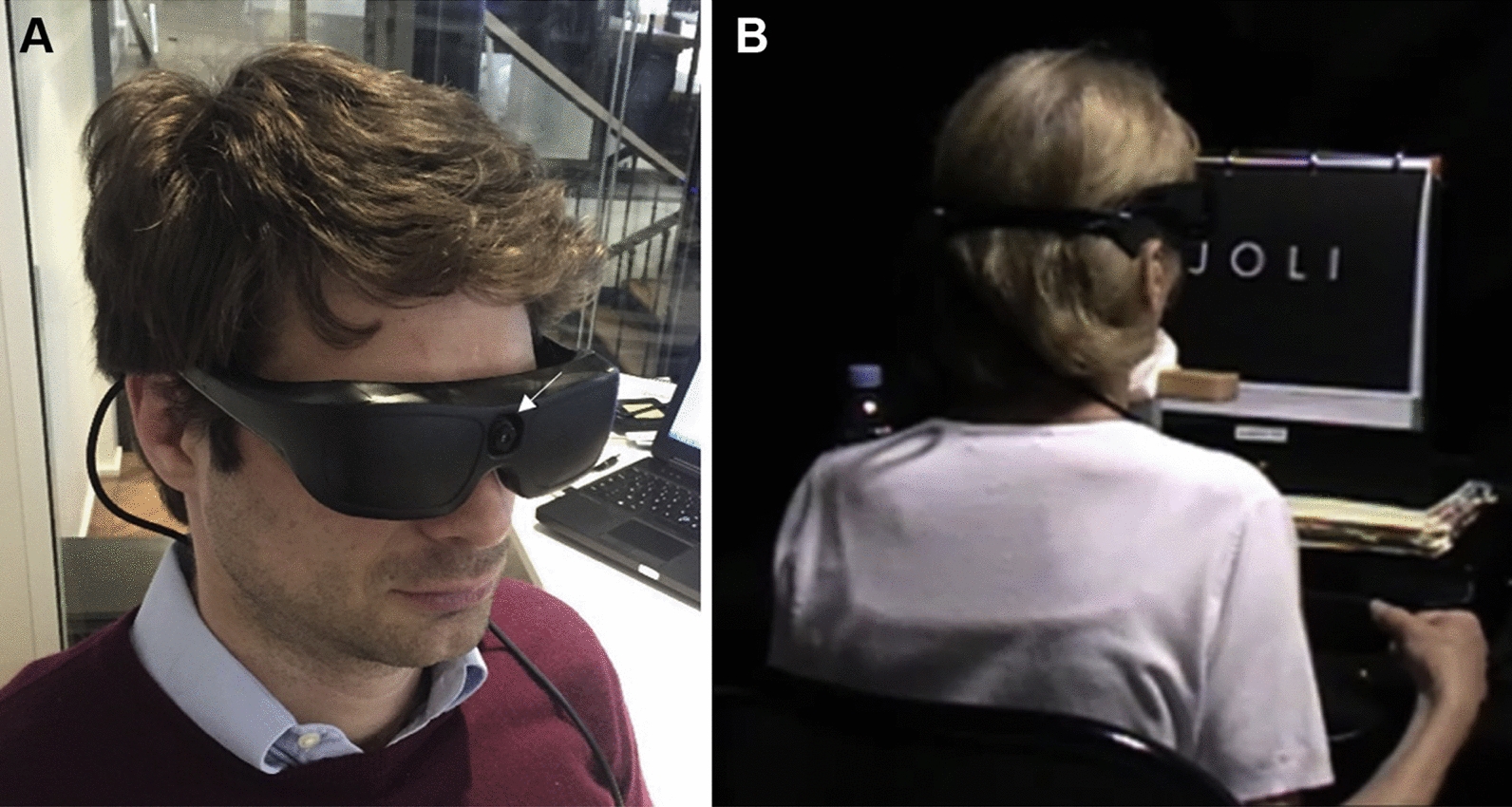
**A:** Photograph showing the opaque video glasses with an integrated camera (white arrow) used in the feasibility study. **B:** Photograph showing letter recognition and reading tests with one of the patients, using the camera mode (Reprinted with permission from Palanker D, Le Mer Y, Mohand-Said S, Muqit M, Sahel JA. Photovoltaic Restoration of Central Vision in Atrophic Age-Related Macular Degeneration. Ophthalmology. 2020 Aug;127(8):1097–1104.)

**Fig. 6:**
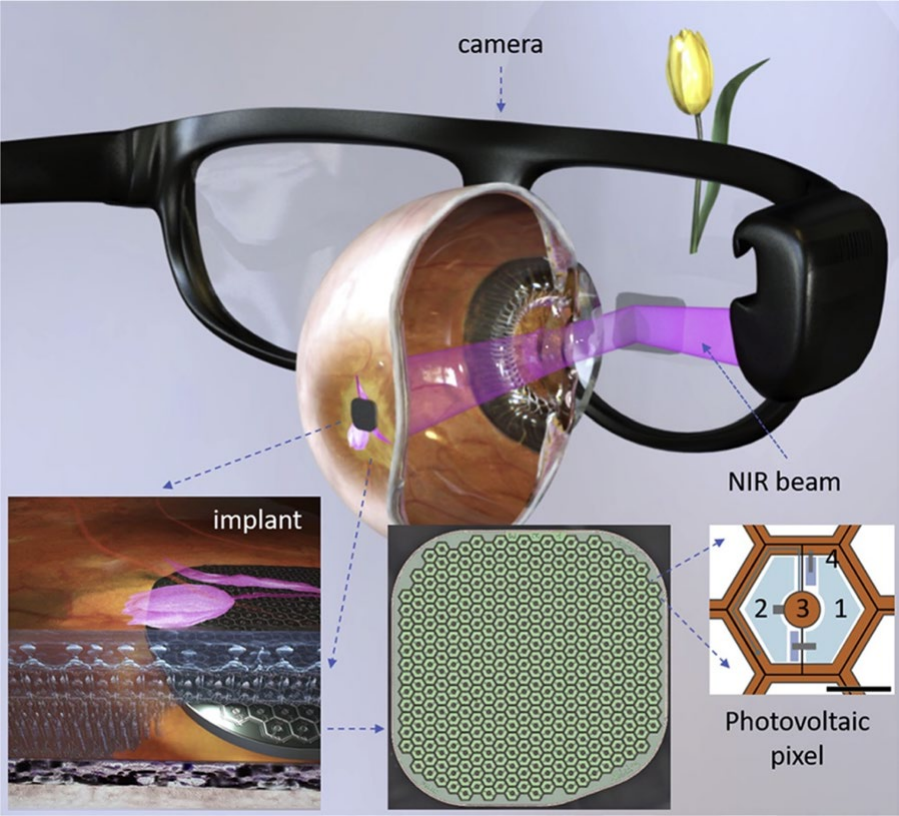
System diagram showing the photovoltaic retinal prosthesis, including the camera integrated into augmented reality-like video glasses, with the processed image projected onto the retina using pulsed near-infrared (NIR) light. Subretinal wireless photovoltaic array converts pulsed light into pulsed electric current in each pixel to stimulate the adjacent inner retinal neurons. Each pixel includes 2 diodes (1 and 2), connected in series between the active (3) and return (4) electrodes. Scale bar 1⁄4 50 mm (Reprinted with permission from Palanker D, Le Mer Y, Mohand-Said S, Muqit M, Sahel JA. Photovoltaic Restoration of Central Vision in Atrophic Age-Related Macular Degeneration. Ophthalmology. 2020 Aug;127(8):1097–1104.)

**Fig. 7:**
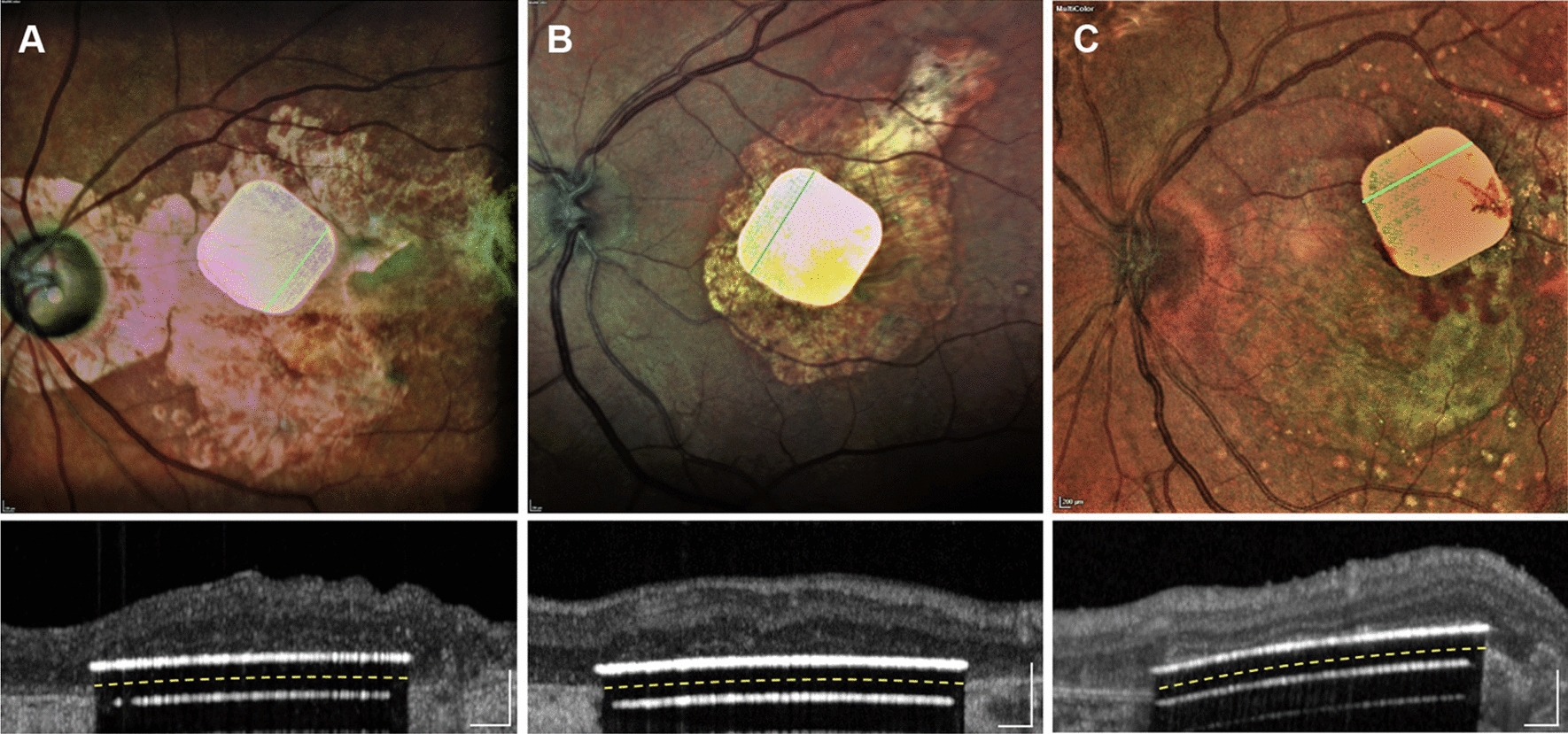
Fundus photographs and OCT images with 3 implants in intended locations: **A:** patient 2, **B:** patient 3, and **C:** patient 5. Images were obtained during the 6-week to postoperative visits (Reprinted with permission from Palanker D, Le Mer Y, Mohand-Said S, Muqit M, Sahel JA. Photovoltaic Restoration of Central Vision in Atrophic Age-Related Macular Degeneration. Ophthalmology. 2020 Aug;127(8):1097–1104.)

The *Photovoltaic Retinal Implant (PRIMA) bionic vision system* is completely wireless and uses photovoltaic stimulation pixels to convert pulses of light into electric current [19, 20]. The design includes a mini camera mounted on a pair of glasses (Figs. 4 and 5) used to capture the visual scene in the environment in order to extract useful information from the images. A miniaturized projector wirelessly projects the images on the internal *PRIMA* implant using near-infrared light (Fig. 6). The photovoltaic cells convert optical information into electrical stimulation to excite the nerve cells of the retina and induce visual perception. Bright pulsed illumination is provided by image projections from video goggles using near-infrared light. Light emits onto a photovoltaic subretinal prosthesis, where silicon photodiodes in each pixel receive power and data directly through the pulsed near-infrared illumination and electrically stimulates the neurons (Fig. 7). The *PRIMA* is currently undergoing clinical trials. As of now, 5 subjects, 60 years and older suffering from dry age-related macular degeneration have received the implant. Performance and safety of the device will be monitored for 36 months. It is estimated to be completed by December 2023.

*Iridium Medical Technologies HARP4k’s Retinal Prosthesis System (IMTC HARP4k)* is a contact lens-shaped wireless retinal prosthesis. It is the first high-acuity spherical bionic retina ever developed [21]. This device stimulates the bipolar and inner neural cells subretinally, while the interface contacts the choroid and the retina. The IMTC HARP4k device supports the acuity needed for recognizing faces and objects, reading big-print books (font size > 14 pt.), and navigating through the environment. Aspects of the device, such as retinal tissue tolerance and influence, were evaluated using 3D computer models and trials on Lang-Yu minipigs. The results of the computer simulation demonstrated that the mechanical stresses exerted on the retinal tissue was within the retinal elastic limit and the tearing energy beneath the retina/RPE adhesion energy. Optical Coherence Tomography (OCT) imaging showed that the retina maintained expected thickness without cyst formation and remained well attached to the chip. Fluorescein Angiography (FA) and Indocyanine Green Angiography (ICGA) demonstrated no sign of vessel leakage during the post-implant interval observed. The study indicated that the *IMTC HARP4k* implant size was within the retinal tolerance and the device is currently undergoing further development [22].

### Suprachoroidal devices

Suprachoroidal implants are surgically placed between the sclera and the choroid of the eye. Devices in this space have the advantages of being less invasive and more easily convenient for repair as implantation of the devices does not require transvitreal surgery. Arguably one of the most significant risk factors is hemorrhage due to the high vascularity of the choroid. Additionally, a large stimulation power is required to evoke visual perceptions due to its location. A major challenge in creation of a reliable suprachoroidal device lies within its increased distance from the retina [18]. [See Table 4: Suprachoroidal device characteristics].

**Table 4:** Suprachoroidal device characteristics

Device	Electrode specifications	Size	Advantages	SAE/AEs	Clinical Status
Gen 2 suprachoroidal device	44 active electrodes (44 × 1 μm diameter)	19 × 8 mm	- Decreased surgical complexity - Less risk of intraoperative and post operative complications - All subjects demonstrated improvement on localization tasks with device on - Expected to be suitable for at home use	No device-related SAEs	Completed clinical trial with 4 patients with RP showing to be suitable for long-term use in humans with RP
Phoenix-99	98 stimulation electrodes and one returning electrode	18.7 × 10.8 mm nominal thickness of 500 μm	Information not available	- Corneal abrasion/opacity - Corneal ulcer - Swelling - Limited blinking - Red eye - Weeping wound, discharge, light bleeding - Suture related - Dislodged orbital grommet (without erosion or VS movements) - Dislodged orbital grommet with erosion through conjunctiva and VS dislodgement - Retinal haemorrhage - Suspected retinal haemorrhage - Limited eye movements - Elevated IOP ≥ 35 mm Hg - Herniated choroid during array insertion	Completed in vivo safety study
STS	49 electrodes (500 Jim diameter and 500 Jim height)	5.8 × 5.2 × 0.5 mm	- Covers a large visual field - conforms to the curvature of the eye - long term use	- moderate edema and hematomas observed in periorbital and head regions - Conjunctival chemosis and injection observed in all cases	Completed in vivo study of wide-field dual-array STS prosthesis

**Fig. 8:**
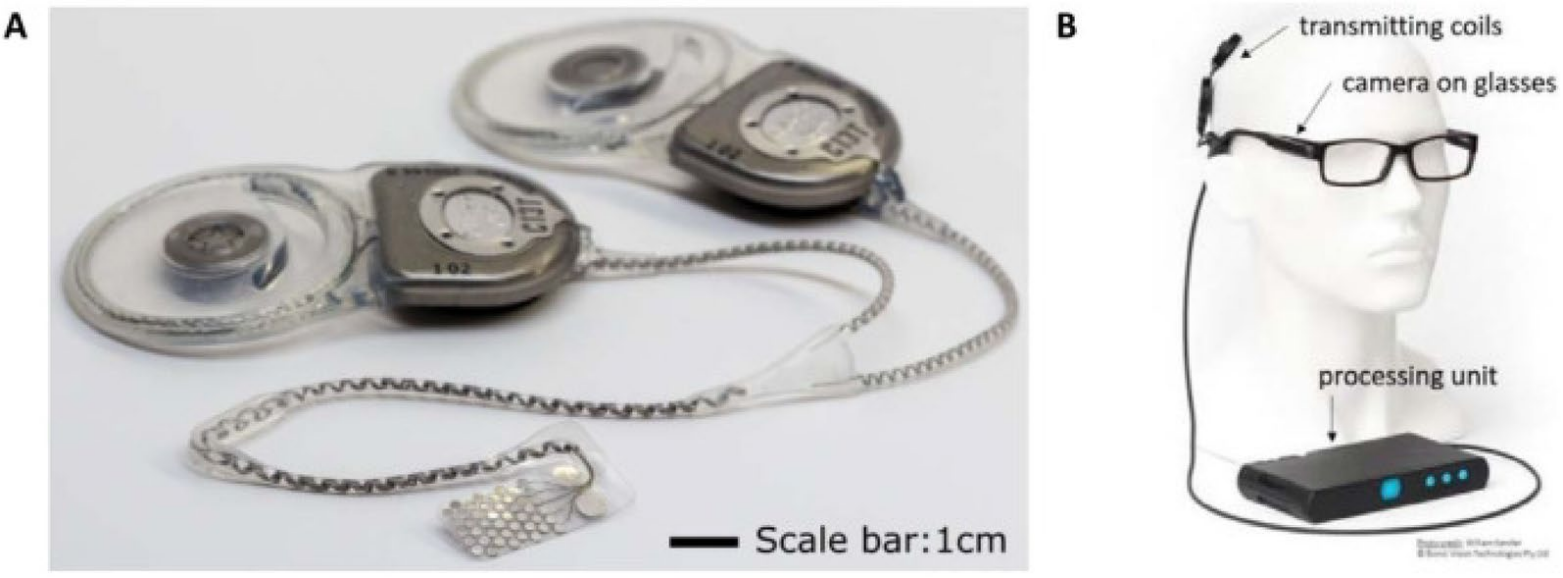
The Generation 2 by Bionic Vision Australia (BVA) **A:** Internal components **B:** External components (Reprinted with permission from Petoe MA, Titchener SA, Kolic M, Kentler WG, Abbott CJ, Nayagam DAX, et al. A Second-Generation (44-Channel) Suprachoroidal Retinal Prosthesis: Interim Clinical Trial Results. Transl Vis Sci Technol. 2021;10(10):12.)

The *Generation 2 by Bionic Vision Australia (BVA)* (Fig. 8) contains an internal 44 platinum disc electrode array each of 1 mm exposed diameter, arranged in a staggered grid in the leading foveal segment. The external component consists of a head piece, spectacles and body piece. Electrical stimulation of the electrodes is achieved by two current sources implanted postauricularly under the scalp. The visual environment is captured by a semiconductor video camera mounted on the pair of spectacles and processed into signals by a video processor located in the body piece. The *Generation 2* differs from its predecesssor (the *Generation 1)* due to the increase in electrode diameter from 0.6 mm to 1.0 mm, decreasing the stimulation charge density. A phase II clinical trial conducted on four subjects who got the device implanted demonstrated no serious adverse events (SAEs) with significant improvements in screen based, functional and avoidance assessments with the device turned on. Additionally, 98% of electrodes remained functional 56 weeks after the initial switch on[23]. Results from this study support the device provides significant improvements in functional vision, activities of daily living, and observer-rated quality of life. Currently, the device is undergoing further modifications in hopes of adopting a device that provides a higher visual acuity.

The *Phoenix-99* is a 99-channel device that is fully implantable. The design of this device utilizes a dual monopolar and hexapolar stimulation pattern that is said to undertake the challenge of retinotopic discrimination and high stimulation thresholds [24]. *The Phoenix-99* has 98 stimulation sites complemented by one common return electrode. The device includes a suprachoroidal electrode array, a visual stimulator and a telemetry implant. The most recent study included the implantation of nine passive *Phoenix-99* bionic eyes in an ovine eye model for up to 100 days.The absence of infection, neovascularization, or histological evidence of tissue degeneration demonstrated biocompatibility of the device. Few SAEs and 41 clinically relevant adverse events (AEs) were observed. Inner retinal layers, particularly the retinal ganglion cell (RGC) layer, were preserved adequately. The system was well tolerated in the ovine model, representing a step towards its clinical potential in restoring vision [24]. Efforts are currently being directed towards seeking ethical approval for in-human clinical trials.

*Suprachoroidal-transretinal stimulation (STS)* consists of a 3D 49-microelectrode array implanted into the scleral pocket. Such positioning was identified to be effective in reducing retinal damage [25]. In a pilot study of two patients using a prototype nine-electrode implant, it was observed that a visual stimulus could be reproducibly elicited in the visual field corresponding to the implant during direct stimulation. Both patients regained the ability to identify and discriminate objects, while one patient was significantly able to detect motion and perform grasping tasks better than by chance [26]. Following the surgical success of both single and dual 49-electode arrays in animal models, three patients underwent implantation of this second-generation device. This time around, functionality tests were found to be less consistent. One subject could localize a square better with the device on during all the follow-up, while two subjects were able to walk along a white line and recognize an everyday object better than chance, but not reproducibly at separate time points [27]. However, the safety profile of the device was found to be reassuring, with no SAEs requiring further surgery after 1 year. Further investigation is warrtanted to draw firmer conclusions about the efficacy of suprachoroidal and transscleral implants in their present formats; however, results to date suggest greater limitations to this approach than for epiretinal or subretinal implants [18].

### Cortical devices

**Fig. 9:**
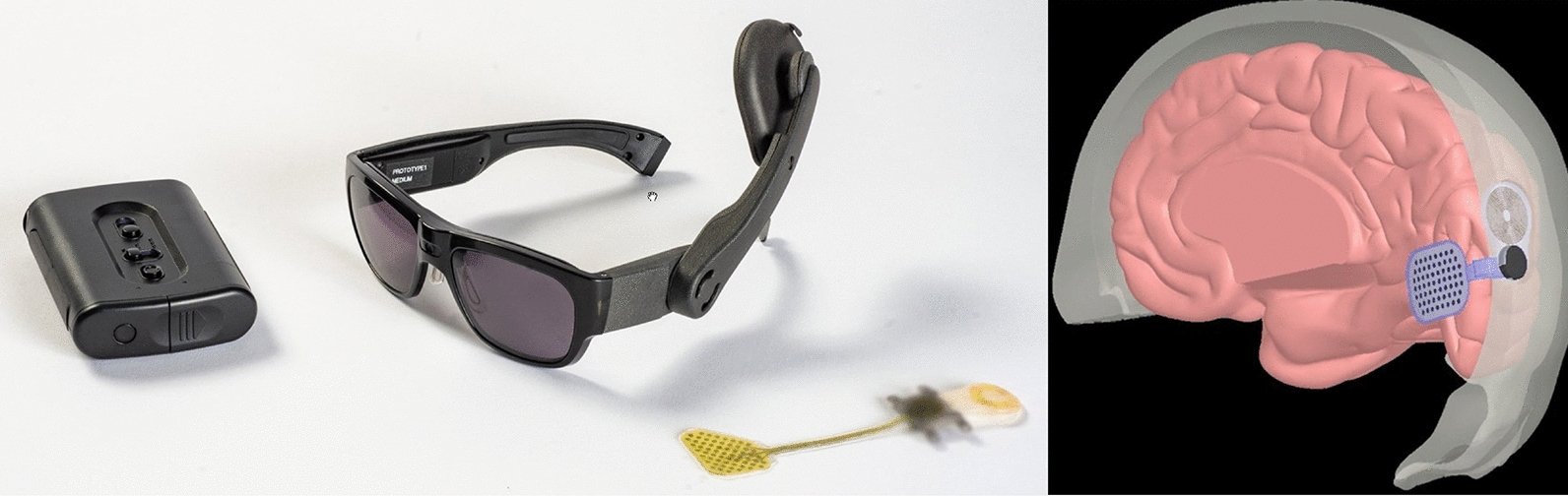
The ORION Visual Cortical Prosthetics system. External components (Left). It is placed on the medial occipital lobe over V1 and V2 (Right) (Courtesy of Gislin Dagnelie, Ph.D.)

*ORION Visual Cortical Prosthetics system* (Fig. 9) was a device manufactured by Second Sight medical products that was identified as the first in-human cortical stimulation device used in the treatment of blindness. By bypassing the injured eye anatomy, this device sends signals to the visual cortex in the occipital lobe of the brain. The device consists of an external and implanted component. A receiver coil, an internal circuit, and a subdural electrode grid with 60 electrodes on the medial surface of the occipital lobe make up the implantable component. The design draws similarity to ARGUS II, with the obvious difference being the direct stimulation of the visual cortex instead of the retina. Among the benefits of ORION are the potential use in patients with significant inner retina and/or optic nerve degeneration/damage and that it is not affected by corneal or lens opacities. The safety of the device was first demonstrated in one blind patient and later in five patients, who all reported visual perception [28]. There is currently an active clinical trial that includes six subjects with bare light or no light perception in both eyes. The purpose of that study is to evaluate the safety, reliability, and usefulness of the device.

As of August 30, 2022, Second Sight Medical Products has merged with an emerging biopharmaceutical company to become Vivani Medical, Inc and they are currently working on *the ORION II device* [29]. The new device is being investigated on how to bring vision to a variety of causes including: glaucoma, diabetic retinopathy, optic nerve injury, optic nerve disease, cancer, and trauma. The device is designed to bypass diseased or injured eye anatomy and to transmit electrical pulses wirelessly to an array of electrodes implanted on the surface of the brain’s visual cortex, where it is intended to provide the perception of patterns of light. ORION was the first in-human cortical stimulation device for the treatment of blindness and data from the clinical stages is currently being used to further develop the *ORION II* device.

The *Cortical Vision Neuroprosthesis for the Blind (CORTIVIS)* manufactured by Biomedical Technologies, SL is comprised of one or two input cameras, a bio-inspired retinal encoder and the Utah electrode array. The design is implanted at the site of cortical layer 4c (the geniculate innervation target), causing the fewest possible neuronal injuries allowing for primary visual cortex stimulation. An early study in monkeys demonstrated that electrical stimulation of implanted electrodes elicited visual perception. Promising results were obtained based on the safe implantation and high-quality visual cortex recordings. One trial (ClinicalTrials.gov identifier NCT02983370) implanted a 96-electrode intracortical microelectrode array in the visual cortex of a 57-year-old man who was completely blind for 6 months. Single-unit recordings were achievable, and phosphene-eliciting stimulation thresholds were within acceptable limits and remained consistent throughout the trial. Simple patterns of electrical stimulation elicited discernable percepts in the blind patient, which enabled them to distinguish object borders and identify various letters. The short-term outcomes in a single patient are promising. Currently, this company is recruiting participants for a study to determine safety and efficacy of the device.

The *Intracortical Visual Prosthesis by the Illinois Institue of technology (ICVP)* stimulates the brain’s visual cortex through utilization of an assortment of wireless floating microelectrode arrays (WFMA) that allow an avenue of communication between the external camera component and the brain’s cortical vision processing regions. This interaction allows for rough visual perception without full restoration of vision [30]. A prototype of the device containing 25 stimulators with 400 electrodes was successfully implanted in one blind volunteer in early 2022. Data from this study are yet to be published. It is currently in Phase 1 of its human feasibility study to assess visual perception and is actively recruiting participants for implantation.

### Optic nerve stimulation

The *Artificial Vision by Direct Optic Nerve Electrode (AV-DONE)* system is an electrode utilizing device implanted into the optic disc. During trials, electrical stimulation sessions were conducted 9 and 23 months after implantation. Patients were questioned about perception of the phosphenes from the center, being recorded in polar coordinates of the visual field. More than 50% of the tests were positive for perception. The thresholds of phosphene perception were also identified as the stimulation current. Ophthalmologic examinations were performed before implantation and at least every 6 months during the 25 months follow up. No severe complications were reported in this period. The device was found to have shortened the surgical time, minimized damage to the optic nerve fibers, and allowed fixation of more electrodes compared to previous devices. These results are suggestive that the device is safe and may be of benefit to future patients[31]. There have been minimal updates since 2009.

[See Table 5: Neural device characteristics].

**Table 5:** Neural device characteristics

Device	Electrode specifications	Size	Advantages	SAE/AEs	Clinical Status
ORION	60 electrodes	Information not available	- Treats a wider variety of diseases - Neural placement - Ability to navigate the environment	- Seizure - Bilateral Hand twitch - Headache - Visual Aura - Visual Phenomenon	Ongoing clinical trials evaluating the safety of the device and surgery
CORTIVIS	100 electrodes (1.0–1.5 mm in length)	4 mm x 4 mm base	Information not available	Information not available	In clinical trial for severe visual impairment with bilateral visual loss
ICVP	16 electrodes per module	2 mm x 2 mm	- No wires or connectors cross the scalp due to wireless nature - Camera images are communicated directly to brain	Information not available	In clinical trials to test the safety of the ICVP system and the feasibility of eliciting visual percepts in response to electrical stimulation in persons with blindness
AV-DONE	7 stimulation electrodes (50 µm diameter)	- The rod: 100 μm diameter - Cylindrical silicone board diameter: 2.0 mm. of the active tips of the stimulation electrodes is uncoated. The wires run parallel to each other. Scale bar = 1 mm	- Easy access to the optic nerve - Stimulate a wide visual field - Elicit small to large phosphenes using just one stimulating electrode	Information not available	Clinical study completed for 1 patient with RP

### Discontinued devices

As advancements are made in the field of visual prosthetics, it is important to acknowledge the preceding devices. *ARGUS II* manufactured by Second Sight was a surgically implanted device that gained FDA approval in 2013 for the treatment of late-stage RP [32]. Between June 2007 and August 2009, 30 patients were enrolled in the ARGUS II feasibility study. As of 5 years after implantation, evidence favored improvement of visual function with 60% of people not experiencing any device or surgery-related SAEs. Overall, 24 SAEs prevailed among 12 patients with many of the adverse effects being reversible with regular treatment. The most common side effects included conjunctival erosion and hypotony. Overall, patients performed better on the square localization test, orientation and mobility tasks with the device as opposed to without it. This device was discontinued completely in 2019 in favor of the ORION cortical device which is still currently under development. While this device received FDA and CE approval in 2013, studies showed the device had around a 5 year longevity deeming it unreliable for lifetime use. Additionally, efforts are being placed towards further developing devices with sharper visual perception than what was commercially available. Patients currently with the *ARGUS II* implant are at a disadvantage as minimal repair and upgrades to the device are available in the event the device malfunctions [33].

The *Intelligent Retinal Implant System (IRIS II)* device is an epiretinal system developed by Pixium Vision SA (Paris, France). There were 6 SAEs that occurred in 4 patients: tack refixation, ocular hypotony due to leakage from sutures sclerotomy sites, vitreoretinal preretinal traction due to vitreoschisis between the array and retinal surface, right leg phlebitis (likely unrelated to the device), and persistent eye pain. The study demonstrated a significant improvement and benefit of high-contrast square object localization and direction of motion performance. The device was discontinued in 2018, due to halted device operation as intended at approximately 9–12 months post implantation.

The *Alpha IMS* produced by Retina Implant AG. Reutlingen, Germany gained CE approval in 2013 and was quickly discontinued and replaced by its successor the *Alpha AMS* which gained CE approval in 2016***.*** Both devices were designed to be implanted in the layer of degenerated photoreceptor cells in patients with degenerative retinal disease. This device operated by stimulating the bipolar cell layer at the retinal input (Subretinal). Unfortunately, the company dissolved in March 2019. The work will only continue for the AMS within its academic partners (University of Tübingen).

## Discussion

### Future directions

As new advances in research and technology emerge, it has been a point of interest to discover new ways of stimulating different cells and brain regions responsible for restoring sight. The evolution of retinal prosthetics has been significant throughout the years so much so that each new product is serving as a steppingstone to something bigger and greater. Regardless of the challenges encountered in the pursuit of a worthwhile device, over 500 people worldwide have had the opportunity to benefit from these technologies. While it may seem like a small number on paper, we must not discredit the fact that some form of vision and perception is being restored to an individual who otherwise would not have any.

Much of the clinical trials now are focusing on stem cell therapy [34]. As of December 2022, there were twelve active international clinical trials regarding stem cell therapy as a potential treatment for RP and other vision threatening diseases with all trials at various phases. Although the results have been promising, there are still complications associated with this form of treatment such as tumorigenesis, suppression of tumor suppressor genes, and stimulation of oncogenes during the production of these cells. Some stem cell types such as multipotent stem cells have been reported to cause retinal detachment which is very risky in a situation where the retina has already been compromised. Ethics are also a very important aspect of stem cell research further adding to the complexity of this as a viable treatment option.

Transcortical retinal stimulation is also being explored as a potential neuroprotective option for patients with RP. This form of stimulation seems to induce the release of anti-inflammatory and anti-apoptotic factors assisting with survival of the remaining retinal tissues. However, it does not promise a restoration in vision. A commercially available device such as the OkuStim by Okuvision (Germany) has shown promising results with a favorable safety profile.

Organic retinal prosthesis are currently one of the most appealing approaches currently being investigated for neuronal stimulation and offer an adequate alternative to the classic silicon-based devices. Different organic semiconducting polymers and pigments have been extensively investigated and specific organic combinations have shown promising results in terms of excellent functionality, high biocompatibility, stability, and flexibility [35]. So far, the efficiency of this approach has only been validated in animal models.

Another advanced therapeutic modality for RP is gene therapy. Voretigene neparvovec (LUXTURNA, Spark Theraputics Inc, Philadelphia USA) is the first gene therapy to gain FDA approval. This gene therapy demonstrated vision restoration and safety in a clinical trial including 41 patients ranging from ages 4 to 44 years with Leber congenital amaurosis [36]. This disease is caused by mutations in the gene RPE65 which represent 0.3–1% of all RP cases. Voretigene neparvovec can only be administered to patients with viable retinal cells. The most common adverse effects are conjunctival hyperemia, increased intraocular pressure, cataracts, RPE changes, and retinal tears. Several clinical trials of potential gene therapy for RP are currently ongoing.

### Challenges/disadvantages

Although several retinal prostheses have yielded promising results, each has its own advantages and disadvantages. A major challenge in the advancement of retinal prosthetics is the length of time to establish outcomes and difficulty in establishing objective and validated outcome measures [37]. It is unknown what the long-term effects of these devices will be, how long they will safely last, and how long they will remain effective. Furthermore, a lack of funding, along with the company becoming more focused on a visual cortical implant, has resulted in the halt of production by leaders in retinal prosthetics such as ARGUS II. The main reasons for this decision were stated to be secondary to the lack of resources and the limited patient population eligible to receive this treatment compared to a visual cortical implant. Compounding a low population size, potential candidates must be physically and psychologically healthy enough for surgery and post implant rehabilitation. [37]. As retinal prosthesis provides a unique vision that differs from natural vision, candidates require a strong support system, a comprehensive understanding of expected results, additional training, and practice to achieve potential results. Patients may also need to travel significant distances to receive surgery and commonly travel out of state for long periods of time during the pre- and post-surgical processes [38].

As more research is done, it is also unknown what will happen once these kinds of devices are commercially available on the market. Barriers to access secondary to social determinants of health (SDOH) may impede low-resourced RP patients from benefiting from such technologies. While currently there are few studies indicating an increased prevalence of RP in specific marginalized groups, cost and availability poses a barrier for patients to receive this type of equipment. There are many barriers to overcome before these devices become commercially available, and the financial impact must be analyzed to judge the access to care that is implicated with these devices. While these devices are new to the market, there is limited research available regarding the long-term costs to maintain these devices. Many of these devices have been discontinued with people still living with the devices implanted.

### Alternative uses

The promising results from studies on RP have encouraged curiosity in the use of these artificial vision prosthetics in other forms of vision threatening diseases. The ORION II device by Vivani Medical, inc. is currently being studied for its effects in restoring vision in patients with glaucoma, diabetic retinopathy, optic nerve injury or disease, cancer, and trauma.

The PRIMA device and IMTC’s HARP4k Retinal Prosthesis System studies are being conducted with a focus on diseases resulting in the degeneration of photoreceptors, especially Advanced Atrophic Dry Age-related Macular Degeneration. The NR600 and BVA devices have been indicated for patients suffering with RP and age-related macular degeneration. The ICVP system is currently being studied for its use in ocular injury, optic nerve diseases, photoreceptor degeneration, and blindness.

## Conclusion

Thirteen devices were found to be current and were presented and ordered by stimulation location. Six have active clinical trials. Four have been discontinued, including the Alpha IMS, Alpha AMS, IRIS II, and ARGUS II which had FDA and CE mark approval. Future directions have been presented.

This review provides an update of retinal prosthetic devices, both current and discontinued. While some devices have achieved visual perception in animals and/or humans, the main issues impeding the commercialization of these devices include: increased length of time to observe outcomes, difficulties in finding validated meaures for use in studies, unknown long-term effects, lack of funding, and a low amount of patients simultaneously diagnosed with RP lacking other comorbid conditions. Future efforts are headed towards more biocompatible, safe, and efficacious devices.

This review has provided a discussion on the most recent report of many of the vital aspects of retinal prostheses. While there is still progress to be made regarding manufacturing a device that restores full vision to a patient, there is promising data demonstrating there is hope for patients suffering from retinal degenerative diseases.

## Data Availability

Not applicable.

## Abbreviations

RP: Retinitis Pigmentosa
BVA: Bionic Vision Australia
SAE: Serious Adverse Event
AE: Adverse Event
RGC: Retinal Ganglion Cell
STS: Suprachoroidal-Transretinal Stimulation
3D: Three-Dimensional
CORTIVIS: Cortical Vision Neuroprosthesis for the Blind
PDMS: Polydimethylsiloxane
PRIMA: Photovoltaic Retinal Implant
RPE: Retinal Pigment Epithelium
IMTC HARP4k: Iridium Medical Technologies HARP4k
OCT: Optical Coherence Tomography
FA: Fluorescein Angiography
ICGA: Indocyanine Green Angiography
AV-DONE: Artificial Vision by Direct Optic Nerve Electrode
ICVP: Intracortical Visual Prosthesis
WFMA: Wireless Floating Microelectrode Array
IRIS II: Intelligent Retinal Implant System 2
SDOH: Social Determinants of Health
IMIE 256: 256 Channel Intelligent Micro Implant Eye
VCTU: Video Capture and Transfer Unit
VPU: Video Processing Unit
NIR: Near infrared
